# Vocal Cord Dysfunction: A Frequently Forgotten Entity

**DOI:** 10.1155/2012/525493

**Published:** 2012-09-16

**Authors:** S. Campainha, C. Ribeiro, M. Guimarães, R. Lima

**Affiliations:** Pulmonology Department, Centro Hospitalar de Vila Nova de Gaia/Espinho EPE, 4434-502 Vila Nova De Gaia, Portugal

## Abstract

Vocal cord dysfunction (VCD) is a disorder characterized by unintentional paradoxical adduction of the vocal cords, resulting in episodic shortness of breath, wheezing and stridor. Due to its clinical presentation, this entity is frequently mistaken for asthma. The diagnosis of VCD is made by direct observation of the upper airway by rhinolaryngoscopy, but due to the variable nature of this disorder the diagnosis can sometimes be challenging. We report the case of a 41-year old female referred to our Allergology clinics with the diagnosis of asthma. Thorough investigation revealed VCD as the cause of symptoms.

## 1. Introduction

Vocal cord dysfunction (VCD) is a disorder characterized by episodic unintentional paradoxical adduction of the vocal cords, primarily on inspiration, inducing paroxysms of glottic occlusion [1–3]. The resulting variable upper airway obstruction can be evidenced in spirometry, when the patient is symptomatic, as a flattening of the inspiratory curve on the flow-volume loop [2, 4].

Common symptoms of VCD include intermittent shortness of breath, wheezing, stridor, or cough, which may be interpreted as uncontrolled or worsening asthma, leading to unnecessary therapy or step up in medication (if coexistent asthma) [5].

The gold standard for the diagnosis of VCD is direct visualization of the upper airway, but due to the variable nature of this disorder the diagnosis can sometimes be challenging [3, 5].

## 2. Case Report

 A 41-year-old nonsmoking woman, was referred to our Allergology clinic with the diagnosis of asthma. She complained of breathlessness, wheezing, and choking sensation with moderate physical efforts and in periods of greater psychological stress. The symptoms had a sudden onset, lasted for about 5 minutes, and despite being increasingly reported over the last three years they were not responsive to inhaled therapy with formoterol/budesonide 320/9 *μ*g bid and on a rescue basis.

She had a past medical history of snoring (OSAS was formally excluded by polysomnography), obesity, and hemithyroidectomy 15 years prior due to a colloid nodule. No symptoms of rhinitis were described. 

There was no familiar history of asthma or atopy. She had several pets (2 dogs, one parrot, and doves) for many years, but she did not notice any relationship between symptoms and contact with them.

Physical examination was unremarkable. Chest X-ray, plethysmography, diffusion-lung capacity, arterial blood gas analysis, and serum IgE were normal. Cutaneous tests for standard allergens were negative. Bronchoprovocation test with methacoline was also negative (PC_20_ > 8 mg/mL).

**Figure 1:**
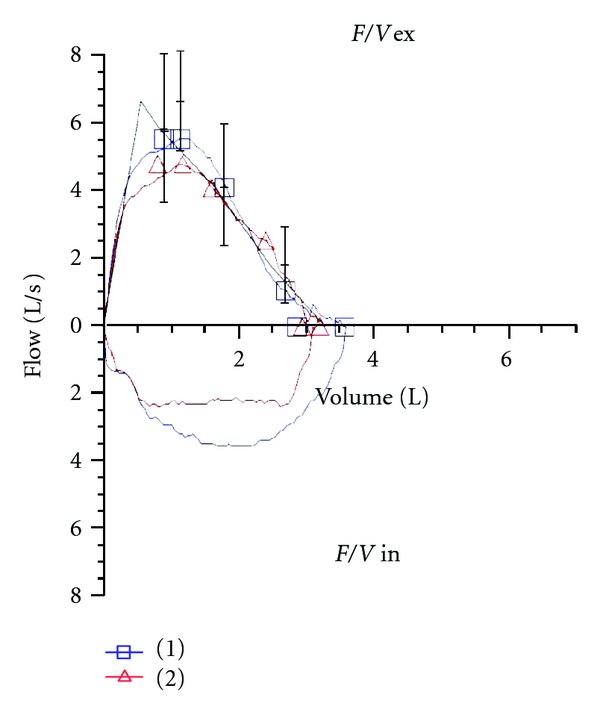
Flow volume loop before (1) and after (2) cardiopulmonary exercise testing (when the patient became symptomatic). An evident flattening of theinspiratorycurve is seen, suggesting avariableextrathoracic obstruction.

The patient underwent a cardiopulmonary exercise testing which disclosed a slight limitation in exercise capacity due to obesity and physical deconditioning. At the end of the test the patient complained of sudden onset of dyspnea and stridor was evident. Spirometry performed during this symptomatic period revealed a flattened inspiratory loop plateau of the flow-volume loop, suggestive of variable extrathoracic obstruction (Figure 1).

**Figure 2:**
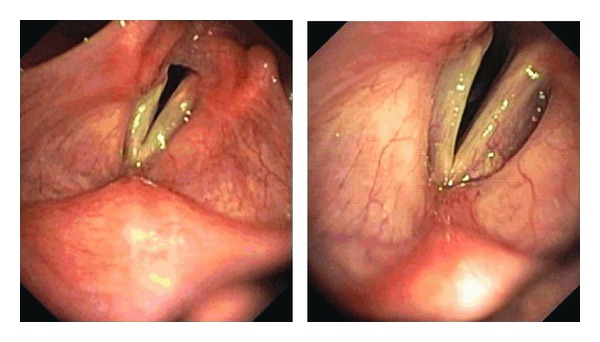
Inspiratory adduction of the anterior portion of the vocal folds with formation of a posterior cleft.

Flexible bronchoscopy performed after moderate exercise showed a paradoxical inspiratory adduction of the vocal cords with a posterior cleft (Figure 2).

The patient underwent speech therapy and psychotherapy sessions with symptomatic improvement. Patient education helped the patient to understand and control her symptoms. Increased exercise tolerance and progressive reduction of day-to-day symptoms of breathlessness and choking was observed.

## 3. Discussion

The true prevalence of VCD is unknown since there have been no prospective cohort studies to assess the development of new cases and many reports lack adequate diagnostic criteria. Several studies report its incidence and prevalence in selected groups, so extrapolation to general population cannot be performed; it is believed to be relatively uncommon [2]. Kenn and Schmitz reported a prevalence of 2.8% of VCD in their prospective study of 1028 patients admitted to a rehabilitation center due to breathing disorders in one year [6]. 

Female gender seems to be more frequently affected, with a reported female-to-male ratio of 2-3 : 1 [7, 8].

The exact pathophysiology of VCD is unclear. Nonorganic (psychological) and organic causes have been reported.

Psychological factors have traditionally been described as an important feature in VCD. Conversion disorder, major depression, obsessive-compulsive disorder, anxiety, and psychiatric disorders have been associated to VCD [9]. Despite being classically described in young physically abused women, it seems like elite athletes, military recruits, and individuals who have had high levels of irritant exposure are at increased risk of having VCD [3]. Emotional stress related to competitive sports seems to be an important trigger.

Exposure to inhaled chemicals such as dusts, fumes, gas, or vapors, postnasal drip and viral upper respiratory infection, can also be a cause or contribute to VCD [2–4, 10]. Laryngopharyngeal reflux, closely related to gastroesophageal reflux disease (GERD) may also play a role on VCD, and 18% of patients with VCD have GERD [8]. Noncompetitive exercise is a commonly recognized precipitant although the exact mechanism is unknown. 

Disorders such as asthma, anatomical, or neurological abnormalities as brainstem compression or movement disorders, laryngomalacia, vocal fold polyps, granulomas or tumors, and unilateral or bilateral vocal cord paralysis in adduction (either tumoral, iatrogenic, or idiopathic) need to be considered in the differential diagnosis [3, 4].

Symptoms of VCD include dyspnea, wheezing, throat, and chest tightness, so VCD is commonly mistaken for asthma. Stridor is frequently present, being loudest above the throat and less audible throughout the chest wall. Dysphonia and aphonia have also been described between attacks of respiratory distress [2]. 

Dyspnea related to VCD has frequently a sudden onset, short duration (<2 min) and is typically self-limited. These symptoms are frequently diagnosed as asthma and are treated for several years with inhaled bronchodilators and even oral steroids. It is not unusual for patients to report a history of recurrent emergency department visits and multiple hospitalizations before the diagnosis of VCD is established [3, 4, 11]. Diagnosis can sometimes be even more challenging if concomitant asthma is observed in a patient with VCD.

Diagnosis of VCD based on symptoms alone is inaccurate. In an asymptomatic period, physical examination will be normal. Chest radiography is typically normal; the features of hyperinflation and peribronchial thickening frequently seen in asthma are usually absent.

During symptomatic period spirometry can demonstrate a pattern of variable extrathoracic airway obstruction on the flow-volume curve (flattening of the inspiratory loop) but its sensitivity and specificity are low. Bronchoprovocation testing with methacholine or histamine, exercise, and specific irritants have been used to induce VCD and detect changes in the flow-volume loop but several studies have shown these tests lack sensitivity [12, 13].

Direct visualization of the upper airway is the gold standard for the diagnosis of VCD. Flexible fiberoptic rhinolaryngoscopy allows evaluation of supraglottic and glottis anatomy, appearance of the laryngeal mucosa, and overall movement of the glottis in order to rule out other etiologies. If performed in a symptomatic period, direct observation will classically show paradoxical inspiratory vocal cord adduction of the anterior two-thirds with a posterior diamond-shaped cleft [2–5]. Greater than 50% inspiratory closure of vocal cords is sufficient for the diagnosis. Depending on the history obtained, provocation with stimuli known to induce symptoms as exercise or cold air can increase the diagnostic yield.

Optimal management of VCD requires the identification of contributing factors and to manage them accordingly. 

Short-term symptomatic control includes relaxation techniques such as pursing lips, panting, and relaxing shoulders. If coexistent, appropriate treatment of asthma is crucial. Anxiolytics are helpful if the patient experiences severe anxiety attacks due to symptoms. Continuous positive airway pressure and heliox mixture were reported to be effective in a small proportion of severely symptomatic patients [8]. Topical lidocaine applied to the larynx and superior laryngeal blocks with *Clostridium botulinum* toxin in selected cases have been attempted with variable success [3, 14].

Long-term therapy includes speech and behavioral treatment. Psychological counseling and psychopharmacologic management are, along with speech therapy, crucial in the treatment of VCD [2–5]. Speech and language therapists play a central role on providing techniques of throat relaxation, cough suppression and throat clearing suppression, and instructing the patient how to control their symptoms. Breathing control techniques also play an important role in the management of symptoms. Inhaled anticholinergics have been reported to be effective in the prevention of symptoms in six patients but further studies are needed to define its role in VCD treatment.

Treatment of coexisting comorbid conditions such as GERD and laryngopharyngeal reflux, rhinitis, or asthma are also an important issue.

Our case report is an example of VCD induced by exercise and psychological factors in a patient who was on asthma therapy without symptomatic improvement. Although VCD is not a frequent entity, it should be kept in mind in order to avoid misdiagnoses and unnecessary and possibly harmful medication. One should not forget that although less frequent, VCD can occur in asthmatic patients. Obtaining a careful history and determining triggers are important in order to establish a correct diagnosis and implement proper treatment.
